# The characteristics of patients frequently tested and repeatedly infected with *Chlamydia trachomatis* in Southwest Limburg, the Netherlands

**DOI:** 10.1186/s12889-020-09334-9

**Published:** 2020-08-14

**Authors:** Juliën N. A. P. Wijers, Nicole H. T. M. Dukers-Muijrers, Christian J. P. A. Hoebe, Petra F. G. Wolffs, Geneviève A. F. S. van Liere

**Affiliations:** 1 Department of Social Medicine and Medical Microbiology, Care and Public Health Research Institute (CAPHRI), Maastricht University Medical Center (MUMC+), PO Box 5800, 6202 AZ Maastricht, the Netherlands; 2 Department of Sexual Health, Infectious Diseases and Environmental Health, South Limburg Public Health Service, PO Box 33, 6400 AA Heerlen, the Netherlands

**Keywords:** Chlamydia, Sexually transmitted infections, Epidemiology, General practice, STI clinic, Repeat infection, Retesting

## Abstract

**Background:**

Repeat *Chlamydia trachomatis* (CT) infections are common. To better understand the characteristics of patients frequently infected with CT at our sexually transmitted infection (STI) care services, we assessed the differences between patients repeatedly infected with CT and those who repeatedly tested negative.

**Methods:**

In this cross-sectional analysis of cohort data, we assessed individuals tested for CT at different STI care providers between 2011 and mid-2018 in Southwest Limburg, the Netherlands (*n* = 17,616). Patients with ≥2 repeat CT infections in the study period were categorized as “patients with repeat CT infections.” Multivariable logistic regression analyses were performed for the binary outcome measure: patients with repeat CT infections versus patients who repeatedly tested negative (reference group). Additional analyses were performed for only the STI clinic population.

**Results:**

Patients aged < 25 years (OR: 1.83; 95%CI:1.38–2.43), co-infected with HIV (OR: 2.07; 95%CI: 1.02–4.22) or co-infected with *Neisseria gonorrhoeae* (NG) (OR: 5.04; 95%CI: 3.33–7.63) had more repeat CT infections. In additional analyses among exclusively STI clinic visitors, patients with urogenital symptoms (OR: 2.17; 95%CI: 1.41–3.35), and patients notified for STIs (OR: 4.55; 95%CI: 3.17–6.54) had more frequent repeat CT infections.

**Conclusions:**

Patients aged < 25 years and patients coinfected with HIV or NG had more frequent repeat CT infections, accounting for ~ 20% of the diagnosed CT infections. These patients are likely at the highest risk for transmitting and acquiring CT. Therefore, testing and retesting this group remains important to enhance CT control.

## Background

*Chlamydia trachomatis* (CT) is a highly common bacterial sexually transmitted infection (STI) worldwide [1]. All patients who test positive for CT should be retested within three to twelve months, according to international testing guidelines [2–4]. It has been shown that up to 32% of the CT patients test positive again within 1 year after diagnosis [5–7].

According to the literature, factor associated with repeat CT infections within 1 year are: having multiple sexual partners, being a woman, patients with a younger age, and coinfection with other STIs, such as *Neisseria gonorrhoeae* (NG) [8–12]. These studies compared the characteristics of patients with one repeat infection to those of patients with no repeat infections. To date, it is unknown whether the characteristics of patients with more than one repeat CT infection over several years differ from those of patients who repeatedly tested negative for CT [9]. Differences in characteristics between patients repeatedly infected with CT and those who repeatedly tested negative for CT could indicate different high-risk populations. However, similarities in the characteristics between these groups could indicate similar sexual networks and high-risk behaviors [13, 14]. Furthermore, the extent to which patients with repeat CT infections account for the total number of diagnosed CT infections could provide insight into CT transmission routes [9]. In an earlier publication, we showed that patients repeatedly infected with NG were mainly men who had sex with men (MSM), HIV positive, CT positive, and diagnosed by the STI clinic [15]. For CT, this could be different since NG mainly affects MSM, whereas CT affects the general population of men, women, and young people (aged < 25 years) [16].

Here, we compared the socio-demographic characteristics of patients with repeat CT infections to patients who repeatedly tested negative for CT. To achieve this, we performed this cross-sectional analysis of cohort data including all CT consultations of different STI care providers in a defined geographical area.

## Methods

### Study design and study setting

**Fig. 1:**
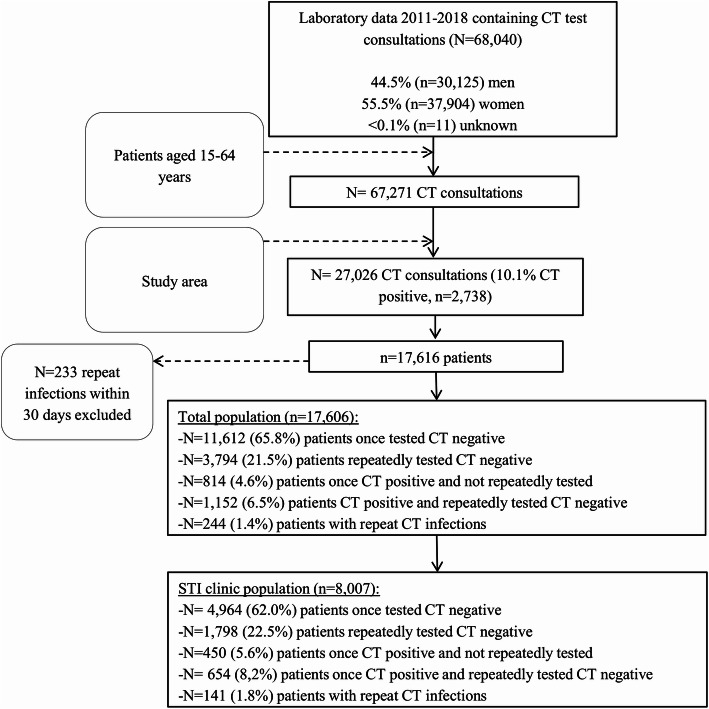
Flowchart of *Chlamydia trachomatis* test consultations, January 2011–July 2018

In this cross-sectional analysis of cohort data, all CT test consultations (*n* = 27,026) of 17,616 patients aged between 15 and 64 years were obtained from the database of the regional Medical Microbiology Laboratory of the Maastricht University Medical Center (MUMC+) between January 2011 to July 2018 (Fig. 1).

### Population and sample size

The database included consultations from all STI care providers of three municipalities (Maastricht, Eijsden-Margraten, and Valkenburg aan de Geul) in the south-western part of Limburg, the Netherlands. The STI care providers were: mental healthcare facilities (*n* = 178; 0.7%), the STI clinic (*n* = 12,170; 45.0%), the hospital (*n* = 3667; 13.6%), and GPs (*n* = 11,011; 40.7%). In the study area, 81% (*n* = 48) GPs sent CT samples for testing to the regional laboratory, ensuring sufficient laboratory coverage [7]. Samples tested positive for CT within 30 days of a previous positive CT test were excluded due to possible false-positive results [4] (*n* = 233). The vast majority of excluded tests (86.7%; 202/233) were diagnosed within 14 days of a previous CT infection. The study area included 111,162 inhabitants (hereafter referred to as the “residential population”) aged between 15 and 64 years (Statistics Netherlands: https://www.cbs.nl/en-gb). We calculated the proportion of residents who were tested for CT once, and repeatedly infected with CT.

### Data management

The dataset was aggregated on a patient level. This means that every patient occurred once in the dataset and all repeat tests and CT infections were added up per patient. Patients who had ≥2 CT infections in the entire study period were classified as “patients with repeat CT infections” (*n* = 244). The majority of these patients had one repeat CT infection (82.8%, *n* = 202), 14.3% (*n* = 35) had two repeat CT infections, 2.0% (*n* = 5) had three repeat CT infections, and 0.8% (n = 2) had four repeat CT infections.

Patients who never tested CT positive and who had multiple CT negative repeat tests within the entire study period were classified as “Patients repeatedly tested negative” (*n* = 3794). The majority of these patients (65.8%, *n* = 2497) tested CT negative twice, 20.8% (*n* = 791) had three negative CT tests, 7.6% (*n* = 288) had four negative CT tests, 2.9% (109) had five negative CT tests, and 2.9% (*n* = 109) and had six to sixteen negative CT tests.

The binary outcome measure was: “*repeat CT infections*,” including patients with ≥2 repeat CT infections within the study period versus patients who repeatedly tested negative (reference group).

### Ethics

The Medical Ethics Committee of the Maastricht University Medical Center (Maastricht, the Netherlands) approved this study (METC 2017–0251) and waived the need for patient consent. Since the retrospective data originated from regular care and were analyzed anonymously, no further informed consent for data analysis was obtained.

### Statistical analyses

In our main analyses, we assessed whether the characteristics of individuals classified in the above described outcome measure were different compared to individuals who repeatedly tested negative (≥2 negative CT tests) using multivariable logistic regression analyses. The determinants analyzed were the initial test location (mental healthcare facilities, STI clinics, hospitals, or GPs), sex (men or women), age (< 25 years and ≥ 25 years), urbanization (rural or urban), HIV coinfection (not tested, yes, or no), and NG coinfection (not tested, yes, or no). All determinants were recorded at the time of the patient’s consultation. We noticed that 6.2% (*n* = 1090) of patients who tested for CT changed their STI care provider after their initial test (Supplementary Table S1). Therefore, the determinant “initial test location” was based on the STI care provider where the patient was initially tested.

Additional determinants were available for the STI clinic population only. Therefore, our secondary study population included only STI clinic visitors tested for CT (*n* = 8007). Additional determinants included the maximum number of sex partners in the past 6 months prior to a consultation a patient had in the entire study period (unknown, 0–1, 2–3, and ≥ 4), any urogenital symptoms during the study period (unknown, yes, or no), any proctitis during the study period (unknown, yes, or no), any oropharyngeal symptoms during the study period (unknown, yes, or no), any notifications of STI during the study period (unknown, yes, or no), and transmission group (men who have sex with women [MSW] or MSM, and women).

Baseline characteristics were tested using chi-square tests. Determinants with *P* < 0.10 in the univariable logistic regression models were included in the multivariable model. All the determinants included in the tables were associated with the outcome measure of interest in univariable analyses and were adjusted for in multivariable analyses. Odds ratios and 95% confidence intervals (CIs) were calculated and presented. All analyses were performed using the SPSS v.24 (IBM SPSS Statistics for Windows, IBM Corporation, Armonk, New York, USA). A *P*-value of < 0.05 was considered statistically significant.

## Results

### Baseline characteristics

The baseline characteristics of the total study population are described in Table 1. Patients with repeat CT infections were more often diagnosed at the STI clinic (57.8% vs. 47.4%; *p* = 0.004), younger than 25 years of age 51.6% vs. 39.0%; *p* < 0.001), HIV positive (4.9% vs. 1.9%; *p* < 0.001), and NG positive (15.6% vs. 3.4%; *p* = 0.001).

**Table 1:** Baseline characteristics of patients repeatedly infected with CT versus patients repeatedly tested CT negative

	Repeatedly tested CT negative	Repeat CT infections	*P*-value
	% (n)	% (n)	
Overall % (n)	100 (3794)	100 (244)	
**Initial test location**			**0.004**
Mental healthcare	0.8 (29)	0.0 (0)	
STI clinic	47.4 (1798)	57.8 (141)	
Hospital	11.2 (425)	6.1 (15)	
General practitioner	40.6 (1542)	36.1 (88)	
**Sex**			0.315
Men	33.3 (1265)	36.5 (89)	
Women	66.7 (2529)	63.5 (155)	
**Age**			**< 0.001**
< 25 years	39.0 (1480)	51.6 (126)	
≥ 25 years	61.0 (2314)	48.4 (118)	
**Urbanization**			0.243
Rural	30.1 (1140)	33.6 (82)	
Urban	69.9 (2652)	66.4 (162)	
**HIV positive**			**< 0.001**
Not tested	28.9 (1096)	21.3 (52)	
Yes	1.9 (72)	4.9 (12)	
No	69.2 (2626)	73.8 (180)	
**NG positive**			**< 0.001**
Not tested	1.1 (43)	0.4 (1)	
Yes	3.4 (128)	15.6 (38)	
No	95.5 (3623)	84.0 (205)	

Statistically significant (*p* < 0.05) associations are depicted in bold

Abbreviations: CT, *Chlamydia trachomatis*, NG, *Neisseria gonorrhoeae*; HIV, Human immunodeficiency virus; STI, sexually transmitted infection

#### CT testing and positivity in the residential population

Of the 111,162 individuals residing in the study area, 15.8% (*n* = 17,616) were tested for CT and 2.0% (*n* = 2210) tested positive at least once; 1.8% (*n* = 1966) were diagnosed with one CT infection, and 0.2% (*n* = 244) with ≥2 CT infections. Within the individuals tested for CT (n = 17,616), 1.4% (n = 244) were repeatedly (≥2) infected with CT. All 2210 CT patients contributed to 2505 CT infections. Of these 2505 CT infections, repeat CT infections (≥2 CT infections) accounted for 21.5% (*n* = 539).

#### Characteristics of patients with repeat infections

##### Total population

Patients repeatedly infected with CT (≥2 CT infections) accounted for 1.4% (*n* = 244) of all the 17,616 individuals tested for CT (Fig. 1). In multivariable analyses, patients repeatedly infected with CT were more likely aged < 25 years, coinfected with HIV, or coinfected with NG compared to patients repeatedly tested negative for CT (Table 2).

**Table 2:** Primary analyses among the total population tested for *Chlamydia trachomatis* between January 2011 and July 2018 including determinants associated with repeat CT infections using patients repeatedly tested CT negative as the reference group

	All individuals tested for CT	Repeatedly tested CT negative	Repeat CT infections		
	% (n)	% (n)	% (n)	OR (95%CI)	Adj. OR (95%CI)
Overall % (n)	100 (17,616)	100 (3794)	100 (244)		
**Initial test location**					
Mental healthcare	0.7 (124)	100 (29)	0.0 (0)	NA	NA
STI clinic	45.5 (8007)	92.7 (1798)	7.3 (141)	**1.37 (1.04–1.81)**	1.02 (0.76–1.37)
Hospital	14.2 (2506)	96.6 (425)	3.4 (15)	0.62 (0.35–1.08)	0.61 (0.34–1.10)
General practitioner	39.6 (6979)	94.6 (1542)	5.4 (88)	1	1
**Sex**					
Men	36.0 (6350)	93.4 (1265)	6.6 (89)	1.15 (0.88–1.50)	
Women	64.0 (11,266)	94.2 (2529)	5.8 (155)	1	
**Age**					
< 25 years	45.4 (7990)	92.2 (1480)	7.8 (126)	**1.67 (1.29–2.16)**	**1.83 (1.38–2.43)**
≥ 25 years	54.6 (9626)	95.1 (2314)	4.9 (118)	1	1
**Urbanization**					
Rural	31.6 (5559)	93.3 (1140)	6.7 (82)	1	
Urban	68.4 (12,051)	94.2 (2652)	5.8 (162)	0.84 (0.65–1.12)	
**HIV positive**					
Not tested	44.9 (7911)	95.5 (1096)	4.5 (52)	**0.69 (0.50–0.95)**	0.77 (0.55–1.07)
Yes	1.4 (246)	85.7 (72)	14.3 (12)	**2.43 (1.30–4.56)**	**2.07 (1.02–4.22)**
No	53.7 (9459)	93.6 (2626)	6.4 (180)	1	1
**NG positive**					
Not tested	5.6 (984)	97.7 (43)	2.3 (1)	0.41 (0.06–3.00)	0.53 (0.07–3.93)
Yes	2.0 (344)	77.6 (128)	22.4 (38)	**5.25 (3.56–7.74)**	**5.04 (3.33–7.63)**
No	92.5 (16,288)	94.6 (3623)	5.4 (205)	1	1

Statistically significant (*p* < 0.05) associations are depicted in bold

Abbreviations: CT, *Chlamydia trachomatis*, NG, *Neisseria gonorrhoeae*; HIV, Human immunodeficiency virus; STI, sexually transmitted infection; OR, odds ratio; CI, confidence interval; Adj., adjusted

##### STI clinic population

In our secondary analyses among only STI clinic visitors, patients repeatedly infected with CT (*n* = 141) were more likely to have urogenital symptoms or notified of STIs compared to patients who repeatedly tested negative for CT (Table 3).

**Table 3:** Secondary analyses among only the STI clinic population tested for *Chlamydia trachomatis* between January 2011 and July 2018 including determinants associated with repeat CT infections using patients repeatedly tested CT negative as the reference group

	All individuals tested for CT	Repeatedly tested CT negative	Repeat CT infections		
	% (n)	% (n)	% (n)	OR (95%CI)	Adj. OR (95%CI)
Overall % (n)	100 (8007)	100 (1798)	100 (141)		
**Maximum number of sex partners**					
Unknown	2.9 (232)	91.3 (21)	8.7 (2)	2.19 (0.44–10.81)	
0–1	25.7 (2059)	95.8 (207)	4.2 (9)	1	
2–3	42.7 (3415)	92.5 (769)	7.5 (62)	1.85 (0.91–3.79)	
≥ 4	28.7 (2301)	92.2 (801)	7.8 (68)	1.95 (0.96–3.98)	
**Any urogenital symptoms**					
Unknown	4.7 (374)	94.7 (36)	5.3 (2)	1.14 (0.26–4.95)	1.53 (0.28–8.29)
Yes	53.9 (4315)	91.4 (1174)	8.6 (111)	**1.99 (1.30–3.04)**	**2.17 (1.41–3.35)**
No	41.4 (3318)	95.5 (588)	4.5 (28)	1	1
**Any Proctitis**					
Unknown	4.7 (374)	94.7 (36)	5.3 (2)	0.73 (0.17–3.08)	
Yes	8.4 (676)	89.8 (230)	10.2 (113)	1.53 (0.98–2.40)	
No	86.9 (6957)	93.1 (1532)	6.9 (26)	1	
**Any oropharyngeal symptoms**					
Unknown	4.7 (374)	94.7 (36)	5.3 (2)	0.70 (0.17–2.93)	
Yes	10.3 (826)	92.0 (289)	8.0 (25)	1.12 (0.71–1.75)	
No	85.0 (6807)	92.8 (1473)	7.2 (114)	1	
**Any notification for STI**					
Unknown	3.5 (282)	95.1 (39)	4.9 (2)	0.98 (0.23–4.13)	1.09 (0.21–5.66)
Yes	14.0 (1120)	81.5 (268)	18.5 (61)	**4.35 (3.04–6.23)**	**4.55 (3.17–6.54)**
No	82.5 (6605)	95.0 (1491)	5.0 (78)	1	1
**Transmission group**					
MSW	30.2 (2415)	90.9 (341)	9.1 (34)	1.33 (0.88–2.03)	
MSM	11.2 (900)	93.5 (360)	6.5 (25)	0.93 (0.59–1.48)	
Women	58.6 (4692)	93.0 (1097)	7.0 (82)	1	

Statistically significant associations (*p* < 0.05) are depicted in bold

Abbreviations: *CT Chlamydia trachomatis*, *NG Neisseria gonorrhoeae*, *HIV* Human immunodeficiency virus, *STI* Sexually transmitted infection, *OR* Odds ratio, *CI* Confidence interval, *Adj*. Adjusted

## Discussion

In this study, we included all CT consultations at STI care providers in a defined geographical area to assess whether patients repeatedly infected with CT were different to those repeatedly tested negative. Patients aged < 25 years or coinfected with NG or HIV more often had repeat CT infections and were likely at the highest risk for acquiring and transmitting CT. Those patients with repeat CT infections accounted for 22% of all diagnosed CT infections.

A comparable study by Hsu et al. showed that 28% of all diagnosed STIs were repeat (≥2) infections [9]. They concluded that a relatively small group of patients repeatedly infected with STIs likely have a disproportionately high impact on circulating STIs within a population; the so called “core group” [9]. The authors included all relevant STIs in their case definition including repeat syphilis, NG, and CT infections. However, we believe that the “core group” theory is STI specific. For example, 76% of the NG infections are diagnosed among MSM in the Netherlands. Whereas CT infections are distributed among different transmission groups (23% of the CT diagnoses are among MSM, 50% among women, and 28% among heterosexual men) [16]. Moreover, heterosexual men, women, and MSM were equally affected by repeat CT infections arguing for population transmission instead of core group transmission (*p* > 0.05; Table 3). Furthermore, 16% of the residential population in our study area was tested for CT. Therefore, only a small fraction of all CT infections was likely diagnosed, leading to ongoing transmission within the population.

Repeatedly infected patients who undergo repeat tests have higher risks for transmitting and acquiring STIs. Repeat CT infections are common among patients who are retested within 1 year (up to 32%) [7, 17–19]. Retesting CT-positive patients is an effective control strategy and can be used to enhance the population-based prevention of CT [4]. Although retesting is advised in many international guidelines [2, 4, 20, 21], retesting rates remain typically low [7, 17–19].

We hypothesized that symptomatic CT patients were more likely to have repeat CT infections. Potentially patients with symptoms are members of sexual networks and at high risk for acquiring and transmitting CT. Hence, STI clinic patients with urogenital symptoms were more likely to have repeat CT infections compared to patients without these symptoms. Our study group and others observed an association between urogenital symptoms and a higher CT bacterial load. This could indicate a higher transmission potential and clinical relevance [22–24]. Furthermore, the highest CT positivity rates are found among patients notified for CT (35.2% in women, 32.8% in heterosexual men, and 22.5% in MSM) [16]. Notably, STI clinic patients notified for STIs were more often repeatedly infected with CT, implying the essential role of partner management for targeting, testing, and treating this high-risk population [25]. Therefore, STI clinics should recommend CT retesting for all CT patients independent of sex or sexual preference.

One strength of this study was that every CT consultation by all STI care providers in a defined geographical area were included to obtain insights in CT testing differences between STI care providers. Due to the inclusion of all tests by the STI clinics and medical specialists and the high coverage of GP data (81%), underestimations of CT tests seem unlikely [7]. Another strength is the timeframe of 7.5 years to partially prevent underestimations of repeat infections [9]. We excluded all positive CT tests occurring within 30 days of an earlier positive test to be able to draw accurate conclusions about patients repeatedly infected, i.e., false-positive test results within 30 days could arise due to non-viable recurrent CT [4]. The additional analyses among STI clinic visitors allowed us to assess additional determinants related to sexual behavior and draw conclusions specified for STI clinic visitors.

One limitation of the study was that information on the reasons for testing was unavailable. Such reasons may include financial reasons. For example, STI tests at the GP are within patients’ deductibles in healthcare insurances, whereas STI tests at the STI clinic are free of charge for risk groups (age *<* 25 years, MSM, and commercial sex workers) [26, 27]. Such reasons for testing could provide additional insight into whether patients were repeatedly tested or not. Furthermore, the additional determinants (sexual risk behaviors and symptoms) assessed in the STI clinic population were not available for the mental healthcare, GP, and hospital populations. We were unable to assess whether patients moved out of the study area or were repeat tested by another STI care provider out of the study area, potentially leading to an underestimation of repeat infections or repeat tests. However, we expect this potential bias to be low, since repeat testing in general at different STI care providers, such as the STI clinic and GP, is proven to be low [7, 17, 19]. A general limitation of studies using existing databases is that data are only available for the tested population, and likely a proportion of all CT infections remain undiagnosed and are not considered. However, this study is one of the few to include all tests by all care providers in one geographical area, therefore including at least almost the whole tested population.

## Conclusions

Patients aged < 25 years, and patients coinfected with HIV or NG had more repeat CT infections accounting for one out of five diagnosed cases of CT. Also, patients treated at STI clinics with urogenital symptoms and notified for STIs had a greater frequency of repeat CT infections. Those patients are likely at the highest risk for transmitting and acquiring CT. Therefore, testing and retesting this group remains important to enhance CT control.

## Supplementary information

**Additional file 1 Supplementary Table S1.** Patients who changed from STI care provider after initial test, 2011–2018

## Data Availability

Due to the Dutch law of protection of personal information (wet bescherming personengegevens Wbp or personal Data Protection Act: http://wetten.overheid.nl/BWBR0011468/geldigheidsdatum_13-07-2015), it is not allowed to distribute or share any personal data that can be traced back (direct or indirect) to an individual. The *Chlamydia trachomatis* data used in our study are third-party data, which cannot be traced back to an individual. The data used in this study are not publically available. For permission, interested researchers are required to provide their name and institution to avoid misuse of this sensitive data and to align with the Dutch law of protection of personal information. Therefore, interested researchers may contact the head of the data-archiving (Helen Sijstermans: Helen.sijstermans@ggdzl.nl) to receive the data.

## Abbreviations

CT: *Chlamydia trachomatis*
NG: *Neisseria gonorrhoeae*
MSM: Men who have sex with men
GPs: General practitioners
MUMC+: Maastricht University Medical Center
MSW: Men who have sex with women
CIs: Confidence intervals
