# Supplementary material for: The characteristics of patients frequently tested and repeatedly infected with Chlamydia trachomatis in Southwest Limburg, the Netherlands
Source: BMC Public Health. 2020 Aug 14;20:1239. doi: 10.1186/s12889-020-09334-9 (PMC7427848; doi:10.1186/s12889-020-09334-9)
Supplement: Supplementary file 1 — Additional file 1 Supplementary Table S1. Patients who changed from STI care provider after initial test, 2011–2018 [file 12889_2020_9334_MOESM1_ESM.docx]

**Title:** The characteristics of patients frequently tested and repeatedly infected with *Chlamydia trachomatis* in Southwest Limburg, the Netherlands

**Authors:** Juliën N.A.P. Wijers, Nicole H.T.M. Dukers-Muijrers, Christian J.P.A. Hoebe, Petra F.G. Wolffs, Geneviève A.F.S. van Liere

Supplementary Material

**Supplementary Table S1.** Patients who changed from STI care provider after initial test, 2011-2018

| **Changed from STI care provider after initial test** | *Chlamydia trachomatis*  % (n) |
| --- | --- |
| Not changed from STI care provider | 93.8 (16,526) |
| From STI clinic to GP | 1.9 (341) |
| From GP to STI clinic | 0.9 (156) |
| From GP to hospital | 1.5 (269) |
| From hospital to GP | 1.0 (185) |
| From STI clinic to hospital | 0.5 (91) |
| From hospital to STI clinic | 0.2 (35) |
| From mental health care to GP | <0.1 (3) |
| From GP to mental health care | <0.1 (3) |
| From mental health care to STI clinic | <0.1 (2) |
| From mental health care to hospital | <0.1 (3) |
| From STI clinic to mental health care | <0.1 (2) |
| Total: | 100 (17,616) |
